# A mixed method analysis of the Botswana schistosomiasis control policy and plans using the policy triangle framework

**DOI:** 10.1186/s41256-023-00321-2

**Published:** 2023-09-06

**Authors:** Kebabonye P. Gabaake, Don Eliseo Lucero-Prisno, Olekae T. Thakadu, Nthabiseng A. Phaladze

**Affiliations:** 1 Department of Medical Sciences, School of Allied Health Professions, University of Botswana, Gaborone, Botswana; 2 Department of Global Health and Development, London School of Hygiene and Tropical Medicine, London, UK; 3 Faculty of Management and Development Studies, University of the Philippines Open University, Los Banos, Laguna Philippines; 4 Okavango Research Institute, University of Botswana, Maun, Botswana; 5 School of Nursing, University of Botswana, Gaborone, Botswana

**Keywords:** Schistosomiasis, Control policy, Ngamiland, Botswana and health policy triangle

## Abstract

**Background:**

The present goal of the World Health Organization (WHO) 2021–2030 roadmap for Neglected Tropical Diseases is to eliminate schistosomiasis as a public health problem, and reduce its prevalence of heavy infections to less than 1%. Given the evolution and impact of schistosomiasis in the Ngamiland district of Botswana, the aim of this study was to analyze the control policies for the district using the Policy Triangle Framework.

**Methods:**

The study used a mixed method approaches of an analysis of policy documents and interviews with 12 informants who were purposively selected. Although the informants were recruited from all levels of the NTD sector, the analysis of the program was predominantly from the Ngamiland district. Data were analyzed using Braun and Clarke’s approach to content analysis.

**Results:**

The study highlights the presence of clear, objectives and targets for the Ngamiland control policy. Another theme was the success in morbidity control, which was realized primarily through cycles of MDA in schools. The contextual background for the policy was high morbidity and lack of programming data. The implementation process of the policy was centralized at the Ministry of Health (MOH) and WHO, and there was minimal involvement of the communities and other stakeholders. The policy implementation process was impeded by a lack of domestic resources and lack of comprehensive policy content on snail control and no expansion of the policy content beyond SAC. The actors were predominately MOH headquarters and WHO, with little representation of the district, local level settings, NGOs, and private sectors.

**Conclusions:**

The lack of resources and content in the control of environmental determinants and exclusion of other at-risk groups in the policy, impeded sustained elimination of the disease. There is a need to guide the treatment of preschool-aged children and develop national guidelines on treating foci of intense transmission. Moreover, the dynamic of the environmental transmissions and reorientation of the schistosomiasis policy to respond to the burden of schistosomiasis morbidity, local context, and health system context are required*.*

**Supplementary Information:**

The online version contains supplementary material available at 10.1186/s41256-023-00321-2.

## Background

Schistosomiasis remains a public health challenge in the world probably outranked only by tuberculosis and malaria [1] as a cause of morbidity. The control and prevention of diseases often depend on the positive interaction between policy content, context, processes, and actors of the prevention strategies. The annual mortality for Sub-Saharan Africa (SSA) due to schistosomiasis is 280,000 [2]. High transmission areas record 60–80% of School-aged Children (SAC) and 20–40% of adult infections [3]. Over 230 million people are estimated to have contracted schistosomiasis globally, and 200,000 deaths occur annually [3, 4]. The five schistosomal species associated with human disease are haematobium, causing urogenital disease, mansoni, Japonicum, and mekongi causing intestinal disease [5]. Transmission of infection occurs when a person comes into direct contact with infested water containing cercariae after it leaves the snail host [6].

The global policy on prevention and control of schistosomiasis dates to 1950 when attempts to interrupt transmission were focused on the elimination of snails, and SAC formed the primary target. The year 1980, marked the era of widespread use of praziquantel for morbidity control, administered on mass and selected risk groups based on prevalence and infection threshold [7]. The Mass Drug Administration (MDA) of treatment was issued to high-risk groups as preventive chemotherapy [7, 8], and in 2001, the World Health Assembly (WHA) resolution 54.19 endorsed chemotherapy using praziquantel as the main control strategy [7, 9]. In addition, WHA 54.19 called for the promotion of health education and access to clean and safe water and sanitation. The goal of health education was to impact behavior change, which was complemented by the provision of sanitary facilities. In 2012 the World Health Organization (WHO) shifted its approach from morbidity control to the elimination of schistosomiasis as a public health problem, targeted for 2020 and interruption of transmission by 2025 in selected areas [7]. In 2020, WHO issued a road Map for Neglected Tropical Diseases (NTDs) control for the period 2021–2030 [10].

An evaluation of control programs around the world revealed that 72 of the 83 countries evaluated remained endemic to the disease despite coordinated control programs because more emphasis was placed on MDA over snail control [11]. It is worth noting that in the context of a one health approach, recognizing the interconnectedness between the human, snail host, and the environment calls for the revision of the prevention and control approaches of schistosomiasis among sub-Saharan African countries irrespective of the level of endemicity. This calls for a multipronged policy and guidelines for national control programs. Moreover, the complexity of schistosomiasis is embedded in space-specific environmental systems and the dynamic of these systems determines the prevalence and effectiveness of interventions [12].

In the context of Botswana, the need for a clear policy to support NTD interventions was realized soon after the government announced that schistosomiasis was burdensome in some parts of the country following several surveys. Between 1985 and 1993, the country implemented a schistosomiasis control program that was integrated into the primary healthcare services [13] and by 1993 prevalence declined to 6.7% hence the program was discontinued.

However, with the social, economic, political, and cultural complexities [14], achieving the goals of the NTD 2021–2030 Roadmap calls for a clearer understanding of its control policy. Hence, it is critical to understand the evolution and impact of the policy and to draw lessons for future programming. This study therefore aimed at reviewing the Botswana schistosomiasis control policies and interventions with the view to identify bottlenecks and successes toward advances in the elimination of schistosomiasis. The specific objectives of the study were to identify the content, actors, policy context and factors facilitating and impeding the success of the schistosomiasis control policies using policy triangle framework.

## Methods

### Study design

**Fig. 1:**
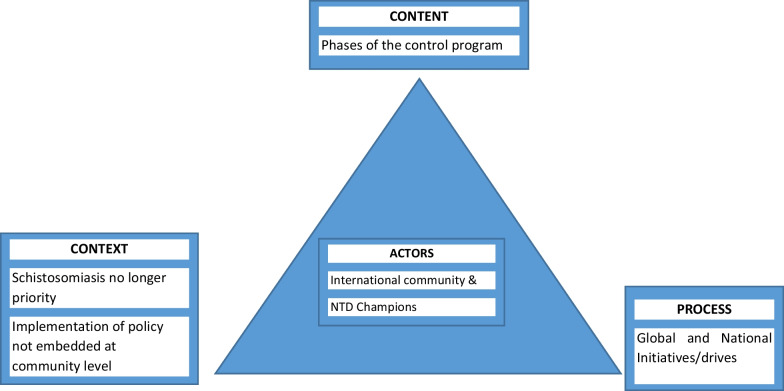
The health policy triangle framework and themes from analysis of interviews. *Source:* Walt and Gilson’s (1994) policy triangle framework adapted to schistosomiasis control

The study design comprised two sequential steps. The first step was an analysis of documents and the second step included in-depth interviews with selected key informants. The health policy triangle (HPT) designed in 1994 by Walt and Gilson [15] was adopted for the analysis of the Schistosomiasis control policy. The triangle model presents a simplified framework based on political and economic perspectives [16] with a paradigm shift in health policy research from a policy content focus to embracing actors, context, and processes (Fig. 1). In our analysis, a review of the policy content comprised an assessment of the subject matter of the policy, its stated intentions, strategies, and operational guidelines to achieve its goal. The policy process assessed how the policy was brought into being and implemented [17]. The actors entailed a review of who the influential participants in a policy process were. An assessment of the systemic factors such as the social, economic, political, cultural, and other environmental conditions influencing the country`s response to schistosomiasis was done to learn about the context.

### The study area and setting

**Fig. 2:**
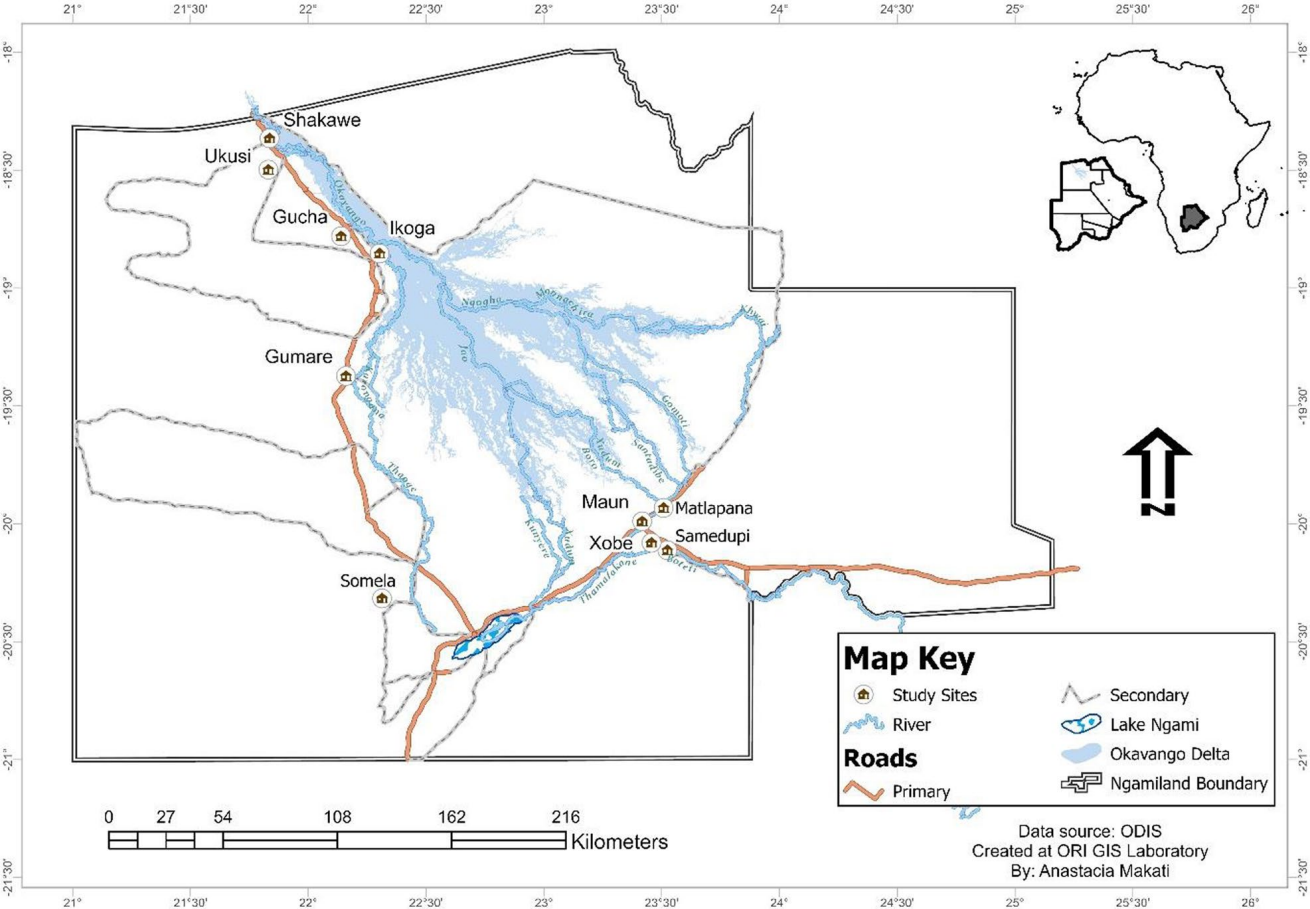
The map of Ngamiland district

The study was conducted in Botswana in the Ngamiland district, located in northwestern Botswana (Fig. 2). The district houses the world’s largest inland delta: the Okavango. Analytic studies have shown that the hydrological system coupled with the presence of snails in the delta has influenced the transmission of schistosomiasis in 1980 [18–21], hence the district was selected as a study site.

### Document analysis

The study used document analysis, in which published documents on schistosomiasis policy (both printed and electronic) were reviewed, synthesized, and summarized. Furthermore, the analysis involved the examination and interpretation of data to elicit meaning and gain an understanding of the policy documents [22, 23]. The rationale for using document analysis was to breed the credibility of our study through the convergence of evidence from the triangulated methods [23]. Moreover, the analysis was used to provide supplementary data, which was used to validate and corroborate findings from the key informant interviews. A document analysis was chosen as time and resources for the study were limited. The analysis of policy documents included a search for articles documenting schistosomiasis control in Botswana. These documents were identified through the MOH website and included NTD strategic plans and published literature relevant to the NTDs control program in Botswana from 1993 to the present date. A thematic analysis where the researcher engaged in the process of pattern recognition within the data, and capturing themes under the elements of the HPT was employed. The reviewed documents are outlined in Table 1.

**Table 1:** List of documents reviewed

Document/report	Year	Policy relevance
Ngamiland Schistosomiasis Control Programme	1985–1993	Documents the prevalence rates, and action plan
The World Health Assembly Resolution 65.21	2012	Provides content on the control of Schistosomiasis
The London Declaration on NTDs	2012	Global Guidance on NTD Control
The NTD Roadmap setting targets	2020	Provides global targets
The NTD master plan for Botswana 2015–2020	2015–2020	Outlined the interventions and Objectives of the control program
The Country NTD Masterplan framework for development	2021–2025	Informed the development of the NTD master plan for Botswana
The WHO Country NTD Master Plan 2021 -2025	2021–2025	Inspired by the review of the NTD master plan for Botswana

### Key informants

A purposive sampling technique was used to select 12 key informants who had first-hand knowledge of NTDS and vector-borne diseases. The researchers adopted Francis and colleagues` approach to determining an adequate sample for qualitative research [24] in which the researchers first specified a minimum sample size for the initial analysis at 9 (initial analysis sample). Secondly, three (3) additional interviews which were conducted without new ideas emerging determined the stopping criteria (data saturation). Informants were first identified through personal and professional networks at the MOH and the University of Botswana. The snowballing technique was used during the interviews for experts to nominate additional informants.

### Data collection

Twelve semi-structured interviews were conducted, ten of which were carried out in English and two in both English and the local language. A semi-structured interview guide was adopted by the researcher from Monnier and colleagues [10]. The guide was then adapted around the components of the HPT to comprise nine questions, 2 for content, 3 for process, 3 for context, and 1 for the policy actors.

The interviews were conducted in person, via Zoom, or by telephone calls to the participants. GPK interviewed all study participants. GPK was a Ph.D. student at the time of doing the research and had prior experience with qualitative interviews through participation in various studies using the same methodologies. Since GPK was not known the participants, GKP introduced herself and then provided information on the objectives of the study before the interview. If the informant agreed, an appointment was set. Written consent for interviews and audio recordings was obtained. Interviews were then audio-recorded and later transcribed for analysis. The researchers took notes, which were used to corroborate and supplement the audio recordings. The interviews lasted on average 45 min. Data collection for this study was carried out between February 2022 and April 2022.

### Data analysis

Braun and Clarke’s [25] six-step approach to thematic analysis was undertaken. Firstly, transcripts were read and re-read for familiarization with the data set (phase 1). This was then followed by phase 2 which involved the generation of initial ideas of what the data is about, leading to the production of initial codes. A codebook (matrix) containing both inductive and deductive codes from the topic in the interview guide was developed (phase 3). The fourth phase involved the sorting of themes and sub-themes from the initial coding system based on the HPT framework. Furthermore, the identified themes were refined, defined, and named to determine the aspects of data each theme captured (phase 5). The themes were then arranged into categories of content, actors, process, and context which comprised the components of the HPT***.*** Verbatim quotes which illustrate each theme were selected based on their representation of the emerging issues [26, 27]. To ensure the trustworthiness of the findings, GPK coded the transcriptions and worked with PN, and DELP during the refinement and naming of themes. The discrepancies which surfaced from the initial codes made by GPK were discussed and resolved during review meetings between GPK, PN, and DELP. Credibility was enhanced through sharing of preliminary findings for validation with three key informants. The consolidated Criteria for Reporting Qualitative Research (COREQ) checklist was used (please see “Additional file 1”). Lastly, (phase 6) the data was put together into a research report.

### Ethics

Ethical approval for the study was obtained from the Ministry of Health and Wellness Human Research and Development Committee letter referenced HPDME: 13/18/1) and the University of Botswana Institutional Review Board (referenced UBR/RES/IRB/BIO/154). Informed consent was obtained from participants, and data were anonymized by using codes. Confidentiality was protected throughout the study, and only the study team had access to the data.

## Results

### Socio-demographic characteristics of the informants

A total of twelve (12) informants participated through modes such as face-to-face interviews (n = 5), online, and telephone platforms (n = 7). Informants were employed at academic institutions (n = 1), government (school, Vector control and Ministry of Health headquarters) (n = 6) community members (village health and Village development committees, political councilor (n = 3), UN Agencies (WHO) (n = 2). Seven (7) of the interviewees had participated in NTD initiatives such as being part of the technical working group for the NTD master plan, developing the NTD strategy, the schistosomiasis mapping exercise and MDA in school and working as officers for the NTD program, and five (5) had collaborated with the WHO NTD Department. Table 2 presents their socio-demographic characteristics.

**Table 2:** Key informants

Key informant	Gender		Educational level	Experience/involvement	Level of involvement
	Male	Female			
Informant #1		✓	Post Graduate	Research	Academic Institution
Informant #2		✓	Post Graduate	Research & Policy Development	National
Informant #3		✓	Diploma	School Policy Implementer	Community School
Informant #4		✓	Diploma	School Policy Implementer	Community School
Informant #5		✓	Secondary	Policy Advocacy in Community Development	Community
Informant #6		✓	Post Graduate	Policy Development and Advocacy	National level- Partner
Informant #7		✓	Post Graduate	Policy Development and Implementation	District Headquarters
Informant #8	✓		Post Graduate	Policy Development and Advocacy	National level- Partner
Informant #9	✓		Graduate	Policy Development and Implementation	District Headquarters
Informant #10	✓		Tertiary	Policy Advocacy in Community Development	Community
Informant #11	✓		Secondary	Policy Advocacy in Community Development	Community
Informant #12	✓		Graduate	Research & Policy Development	National

### Key findings from document analysis

The Ngamiland schistosomiasis control program policy for 1985–1993, the NTD Master plan for 2015–2020 plans, and other significant regional and international protocols and conventions with relevance to the development of the schistosomiasis control program in Botswana were reviewed and analysed. The analysis was done based on content, context, processes, and actors.

Content. The Ngamiland schistosomiasis control program for 1985–1993 had clearly stated objectives, and interventions, and used surveys as monitoring and evaluation tools for the performance of the program. The implementation plans highlighted key interventions, and targets derived from the World Health Assembly resolution 54.19 [1, 28]. The control program was deficient as it omitted content on snail control interventions, health education, and behavior change strategies. Moreover, the focus of the program was primarily on treating the SAC [28] and little attention was paid to other groups at risk of the disease. Our analysis also found that good policy intentions and practice could not be sustained over time because the policy lacked dedicated resources. It is also worth noting that, a decision to use a 10% prevalence as an upper limit for initiating maintenance of control alone without considering treatment coverage rate and continued surveillance, limited control of the disease, hence its resurgence post-1993. After the abandonment of the program in 1993, the MOH officials and partner organizations reviewed NTD global policy documents and cascaded and adopted them to formulate national NTD control plans. The documents which specifically inspired the plans were the NTD Roadmap setting targets for 2020. Adopted from the aforementioned documents, the Botswana NTD Master Plan 2015–2020 was formulated. The plan comprehensively outlines the goal targets and interventions for the control and prevention of schistosomiasis.

Context. The fundamental contextual elements that shaped the development of the schistosomiasis policies for Botswana were the burden of schistosomiasis, donor support and dependence. The review revealed that the schistosomiasis policy formulation was initiated to control morbidity for the disease. The Ngamiland schistosomiasis control program of 1985–1993 would not achieve the morbidity control target without external aid, making development partners' support essential to maintaining service delivery. The NTD master plan came into being to augment the limitations of the national response plan which only focused on high prevalence areas and lacked mapping of NTDs and yet wished to eliminate them. The policies analysed within the context of the country, region, and other international protocols are all relevant in terms of providing contextual guidance for the control of schistosomiasis. The limitation we gathered during the analysis was the lack of domestic funding and resource allocation for policy implementation.

Process. The master plan was formulated after a comprehensive consultative forum led by the MOH, WHO, and the Ministry of Education. WHO led the process by bringing a consultant from the Schistosomiasis Control Initiative (SCI). A pertinent concern with the schistosomiasis policies was that they needed updating. The Ngamiland policy (1985–1993) and the Botswana NTD master plan for 2015–2020, were outdated and not approved. The Ngamiland Schistosomiasis Control Program 1985–1993 has outlived its time, as was formulated more than 3 decades away. There is a need to review and revise these policy documents to align them with any new developments. At the time of the development of the Ngamiland control program, the schistosomiasis mansoni was the most prevalent but the situation has changed to schistosomiasis haematobium as the high-ranking type of disease currently. Moreover, the assessment for NTD strategic plan development showed that no (zero) district was mapped or had a known status of endemicity. In this regard, the policy lacked evidence for its actual formulation.

Actors. The analysed documents resided at the MOH, which removes the challenge of fragmentation. The added advantage of the policies reviewed is that they embraced the multi-stakeholder approach in policy formulation and implementation as espoused by the NTDs control approaches. The Botswana 2015–2020 master plan specifically highlights the important role of multi-stakeholder participation and involvement in policy formulation and implementation. However, the stakeholders for both the Ngamiland policy and the 2015–2020 Master plan were skewed toward the MOH and WHO. There was little representation of other key stakeholders like the MLGRD, water and sanitation, wildlife and tourism, the Ministry of Agriculture, and others. Moreover, the stakeholder analysis revealed the lack of structured representation from a systemic perspective of them coming from national, district, and local level settings. There was minimal participation of local communities, the NGO sector, and the private sector participation in the policy formulation, implementation, and evaluation. Most of the actors were roped in for their technical and financial assistance. This approach negatively impacted the sustainability of the policy interventions.

Table 3 summarizes the results from the informant interviews into five (5) broad themes and sub-themes according to the HPTF. Theme I was about phases of the control program, while Theme II was about schistosomiasis being no longer a priority, Theme III was the implementation of policy not strongly embedded at the community level, Theme IV was global and national initiatives, and Theme V was about the fact that policy was driven by the international community and NTD champions.

**Table 3:** Summary of theme and sub-themes

Component of PTF	Broad themes	Sub-themes
Content	Theme I: Phases of the control program	Morbidity control
		Climate change caused policy agenda to resurface
		A refocus on morbidity
Context	Theme II: Schistosomiasis is no longer a priority	Inadequate resources
	Theme III: Implementations of policy not strongly embedded at the community level	Lack of robust community participation and health promotion
		Lack of knowledge and uptake of services
		Multi-sectoral approach
Process	Theme IV: Global and national initiatives/drive	Integration
		Lack of evidence-based programming
		Lack of vector management strategies
Actors	Theme V: Policy-driven by the international community and NTD champions	Ministry of Health
		Ministry of local government
		Ministry of Education through schools in endemic areas
		WHO
		No local NGOs
		Village committees

#### Theme I: Phases of the control program

The key informant interviews revealed that schistosomiasis was a serious infectious disease in the 1980s. Endemic districts used the terms “Thutisa Madi, thota Madi” meaning blood in urine, to describe the disease. Interviews with informants revealed three sub-thematic phases for the control policies which were;

##### The morbidity phase

This was the phase that placed the control and prevention of schistosomiasis control as a high-level priority, especially for the Ngamiland district where morbidity was high. Cooperation among sectors to combat the disease within the district was developed and the program received support from WHO. According to the informants, the policy aimed at reducing the prevalence of the disease and lessening heavy infections among SAC, and very little attention was paid to other groups at risk of the disease. Key informants also reported that the disease control interventions included diagnostics, treatment, data management, and surveys. A decision to use a 10% prevalence as an upper limit for initiating maintenance of control was set. Key informants stated that this phase was about policymaking for the prioritization of schistosomiasis control in Botswana. The output of this phase was a centralized disease control program mostly operated by the Ministry of Health with technical and financial support from WHO. The morbidity control phase marked a success for the control of the disease as one informant mentioned;

“The past efforts for the program was a success story that Botswana wrote… countries were brought here to come and see what Botswana did” – (Medical entomologist, informant #12).

##### Climate change caused the policy agenda to resurface

Eventual neglect of the control program came in 1993, and it was until 2015 that the mapping of schistosomiasis indicated significant rates. An informant from the government associated this phase with the resurgence of the disease which brought back the disease onto the policy agenda. Unlike other vector-borne diseases, the informants mentioned that schistosomiasis lacks environmental control interventions. In the discussion of the changing dynamics in the transmission of the disease, one informant stated that;

“Rain is occurring at times it never used to, floods are in places where they never appeared, and we are now getting schistosomiasis in communities that were never schisto endemic…. Batswana are a mobile community moving three homesteads with their diseases” - (Program manager, Informant #2)

Furthermore, the key informants pointed out that the lack of targeted interventions during the neglect phase was associated with the negative climate change on the disease. One key informant echoed that:

“Targeting hot spots communities in endemic areas where there are likely environmental changes than doing the historic blanket MDA, was needed for the control of the disease” - (Program manager, informant #1)

##### Refocus on morbidity control

In response to a regrettable phase of neglect of the program, a plan to occasionally map hot spots and conduct country-wide surveillance of NTDs including schistosomiasis was devised in 2015. The mapping survey then revealed a high prevalence of schistosomiasis. According to WHO and MOH informants, the schistosomiasis country policy agenda was revised, to include aspects of the NTD global policy documents, cascading them to formulate national NTD control plans. One informant mentioned that the output was a guideline document on morbidity control and robust and standardized M&E systems. Adopted from the global guidance, the Botswana NTD Master Plan 2015–2020, was developed. The plan comprehensively outlines the Interventions for the control and prevention of schistosomiasis. Accordingly,

“The country is doing its first time MDA countrywide.” (Program manager, informant # 2)

#### Theme II: schistosomiasis no longer a priority

The control program was enacted in 1985 to counter the impact of the disease. The coordination of the program was done by the Ministry of Health and WHO offices. The policy changes which followed were influenced by internal and external factors. When asked what was needed for the implementation of a schistosomiasis interruption policy in Botswana, informants mentioned that many of the control interventions were ineffective, outlining the main contextual challenges of the policy. Moreover, following the attainment of low prevalence thresholds, the government decided not to give schistosomiasis the priority it deserved and consequently failed to come up with policies and surveillance measures supporting sustained control.

One informant lamented that:

“The schistosomiasis program was strong at some stage to the point when the MOH felt they had done away with the disease and thought there was no need to spend energy and resource on it. The MOH never took into account that when you bring a disease to elimination, the program must stay alive so that it tracks the resurgence of the disease….but the disease was allowed to run wild” - (Medical Entomologist, Informant # 12)

The chronic nature of the disease has been associated with the lack of prioritization of the diseases. An informant stated that:

– “When a disease does not cause fatality, people are slow to react, like in the case of schistosomiasis”.-(Policy maker, Informant # 8)

##### Inadequate resources

Consequent to the lack of prioritization of the disease, the national control program was abandoned, and domestic funding, research, involvement, and coordination of actors of different sectors within the NTDs community were neglected as well. Key informants lamented the absence of an organized disease control program which led to a lack of financial resources which an informant described as;

“A speech of the pipeline machinery, without action”. - (Policy marker, Informant #8).

Of importance to note was the lack of a nationwide program where informants stated that;

“Currently the NTD program is nested within the TB program and doesn't have its budget. We depend on partners; and WHO is the only partner currently. So that poses a challenge in terms of expanding beyond school-aged children, because WHO only funds MDAs for SAC. We also are not able to reach the district as frequently as we would like to for surveillance. Staffing is also an issue because the program staff is also TB staff”. - (Program manager, informant #2)

Informants indicated that the water and sanitation situation in the Ngamiland district was generally poor and recommended the engagement of the community towards improving the water and sanitation situation. Expanding on the resource constraint issue, informants explained that creating an NTD label was fundamental to schistosomiasis receiving less attention than other diseases. Another part of the NTD framing argued by key informants was that of the “elimination or eradication of schistosomiases in Botswana in 1993”. According to the informants, the use of the words created a loss of focus for the national control program. An informant explained that:

“The word neglected, simply means we know and we neglected! It's good that we know, and we have since reminded ourselves that there is a need for us to give the disease a new focus on control. We had started very strong and lapsed on the way, hence we started again” - (Medical entomologist, informant #12).

#### Theme III: implementation of policy not strongly embedded at the community level

Community based key informants shared a comparison of the implementation of malaria and diarrhoeal diseases, pointing out that the control activities for the two diseases were strongly enchored on community partnerships citing the sub-themes below:

##### Lack of robust community participation and health promotion

The key informants in the Okavango district lamented the lack of community participation in health education, water, and sanitation measures which in turn hampered the uptake of disease prevention efforts. The need for health education and sanitary facilities was overly emphasized by the community informants. Compared to malaria and other vector-borne diseases, the level of health education for schistosomiasis was low among SAC. A key informant pointed out the need for intensive community education at the clinics, in the homes, in schools, and in “kgotla” *(traditional)* meetings. One informant stated that;

“Since our communities have a lot of water-based activities like fishing, teaching communities about protective clothing when washing clothes at the river is very vital. If the schistosomiasis health personnel could do like malaria and HIV, the people will change their behaviors”. - (VDC/community leader Xakao Village, informant # 5).

##### Lack of knowledge caused poor uptake of services

The District Management Team (DHMT) and community key informants alluded to a lack of knowledge of the disease among communities even in endemic areas. Even though the disease has existed for decades within the district, informants mentioned that the communities lack knowledge because children, fishermen, and farmers wade the waters without protective clothes and still defecate and urinate in rivers. One informant stated:

–*“*If education is given to communities, changes in behavior are realized. Our children have been taught about playing in the water and the danger of crocodiles killing them and they have since stopped, people accept health education and without it, at times negative consequences like using mosquito nets for fishing can result” - (Teacher at school and Community leader, Informants # 4 and 5)

##### Multi-sectoral approach

A multi-sector approach was mentioned as key to the schistosomiasis control policy. In a discussion with a DHMT leader, it became evident that a multi-sectoral approach produced results for the malaria program and its deficiency in the schistosomiasis program brought negative ramifications. Collaboration with environmental health, local authorities, communities, and other sectors of the government and civil society organizations were viewed as important for health education and disease prevention efforts. The informants described the lack of internal and external collaboration; operations and control strategies as “implementation in silos”. An informant shared that*;*

–*“*The DHMT was not aware of schistosomiasis control policies because they were never informed about it” - (DHMT manager, Informant # 7)

#### Theme IV: global and national initiatives

The process of initiating the Schistosomiasis policy was based on the high prevalence of the disease in the 1980s. While several key informants postulated that the MOH led the agenda-setting and exerted the most influence in the policy process, they also noted the support from WHO and the country`s quest to adhere to and reach the global health agenda as push factors for the development of policy plans. Of importance to note were the following sub-theme.

##### Integration

According to key informants in the study, in the early 1980s, the control and prevention program for schistosomiasis was integrated into the existing MOH primary healthcare system, where health education, adequate water supplies, and sanitation were successfully integrated into the healthcare system in Ngamiland. The program was anchored on a strong PHC base which the country had. The integration also involved teaching about the disease in varied health training programs and conducting in-service sessions. An informant from the health training sector alludes that:

“All health workers were aware of the disease, and focal persons were available at district and national levels to do supervisory visits” (Lecturer, Informant # 1).

The key informants reported the integration efforts vanished with the discontinuation of the program after the country attained the set targets of elimination.

##### Lack of evidence-based programming

The discontinuation of surveillance and mapping of disease took place in 1993 when the program focused on testing through Kato Katz and praziquantel treatment for those diagnosed with the disease. A historic account from one key informant revealed that from 1993 to 2015 prevalence estimates were not frequently determined, marking a collapse in surveillance modalities for the diseases. The country formulated and implemented policy changes to catch up with the neglect of the disease. However, the lack of national conversations among NTD champions, and deficient political ownership of the disease were noted as hindrances to the implementation of the policy. Moreover, a key informant explained that;

“It was only in 2015 that the control program was resuscitated through a WHO-funded mapping exercise. This was a response to the WHO elimination targets hence the drafting of the 2015–2020 NTD master plan”. - (Policy Maker WHO, informant #8).

The informant further echoed the need for continued surveillance as a means of generating evidence for the policy and emphasized it by saying;

– “Mapping of the disease is important at all phases of the disease, from control to the pre-elimination and elimination phase, to avoid dancing in a cycle”.- (Medical entomologist, Informant #12)

##### Lack of vector control and management strategies (VCMS)

For a longer duration, the Botswana control program omission of mollusciciding in the control program hindered the control of schistosomiasis and hence the disease resurged. This has not brought sustained interruption of transmission in Botswana. An informant uttered that;

– “The lack of a program at the ministry currently means that the program is only aligning with WHO strategies which could lack the Botswana context. There is a national VCMS but since the NTD program does not have a budget at MOH, its implementation has been limited”. - (Vector control manager, informant #6)

#### Theme V: push from international community and NTDs champions

The major actors in the development of the schistosomiasis policy plans were the MOH and WHO. WHO`s support for the policy process and implementation continued to be the strongest in the control of schistosomiasis in Botswana. The schistosomiasis control program for the period 1985–1990, comprised a multi-sectoral national Schistosomiasis Taskforce which operated a fund from the Enda Mc Connel Clark Foundation of New York and assistance from MOH and technical support from the WHO Geneva office. Since then the program operated on minimal financial support from MOH with WHO expertise, evidence, and global guidance for Botswana to adopt and domesticate. Other Botswana government actors included the then Ministry of Local Government and the Ministry of Education through schools. The communities consumed services at primary healthcare facilities.

## Discussion

This paper provides qualitative insights into the policy content, context, process, and actors in the control of schistosomiasis in Botswana. Over the past years, several resolutions have been made to control schistosomiasis. Drawing from these resolutions, countries aimed at reaching the set targets for morbidity control, and elimination. Regarding policy content, unlike most sub-Saharan African nations [29], our analysis revealed the presence of clear and specific goals, targets, plans, and implementation strategies to address the burden of schistosomiasis morbidity in Botswana. The set targets were realized within specified time frames as they clearly defined what institutions and persons will achieve and in what time frames [1]. Of importance to note was that the strict adherence to the set target led to the success of the control policy [1]. Similar findings were obtained by Collins and colleagues in China [30].

Our analysis highlights that Ngamiland’s health policy failed to include and prioritize snail control as much as MDA. Successful policies in other countries were more elaborate and supported snail control [29, 31, 32] hence managing to block the parasite’s transmission, and thus truly preventing human schistosomiasis over the long term. In addition Perez [33] in his modeling of eco-hydrological aspects of schistosomiasis, highlighted the importance of the inclusion of ecological models in the control and elimination strategies of the disease. Our study records advocacy notes for the elimination of intermediate snail hosts from local habitats and means to prevent water contamination through sanitation similar to Kings and Colleagues [31, 32, 34]. The lack of a monitoring and evaluation and surveillance mechanism was another deficiency that we identified in our analysis of the policy content. The process of transmission of schistosomiasis proved to be very focal and highly efficient in Ngamiland, such that, a resurfacing of the disease was realized after a phase of neglecting to map the disease. It became clear that the MDA cycles limited transmission by reducing environmental contamination with parasite eggs. However, the lack of a VCMS which could determine the impact of aquatic pollution in the transmission of schistosomiasis in Ngamiland, pointed out a collapse in the surveillance of schistosomiasis. Schistosomiasis then assumed a neglected disease tag, which meant missed diagnosis, or even a silent national epidemic without clear guidance on its control. Similar concerns were raised by previous researchers [35, 36]. Our assessment of the policies found an era of the refocus of the control program to align with changes in global standards and targets.

The policy analysis noted national and international contextual influence on the development of the policies. The high prevalence of schistosomiasis prompted the formulation of the Ngamiland 1985–1993 control program. The 2015–2020 master plan was formulated on the contextual background of the lack of known endemicity of the disease and yet the country subscribed to the global call for schistosomiasis elimination by 2030. The study identified an administrative gap in the context of schistosomiasis policy. There was top-down performance management where guidance and regulation of policies were from MOH. However, we noted that an experimental Implementation that is amenable to a bottom–up approach, sensitivity to the implementation context, and support for problem-solving [37] would have been sustainable.

Overall, the policy process was consultative and was driven largely by the global health community, and its scientific evidence. However, our study highlighted the lack of evidence for the development of the Botswana NTD master plan 2015–2020 and the lack of contemporaneousness of both the Ngamiland policy and the master plan. An outdated policy is bound to be non-contextual, irrelevant, and uniformed [37].

Even though the Ngamiland district health team produced policy documents and guidelines which focused on morbidity control, these documents conspicuously lacked an explicit strategy to control the national morbidity of the disease. It is also pertinent to note that the 1985–1993 control policy and the 2015–2020 NTD master plan gave inadequate insight into what intervention packages would be implemented, with what resources, and by which sector. The country depended on funding from WHO which often came in as funds for purchasing drugs for MDA campaigns. This could have contributed to the poor sustainability of the integration of services into existing health systems, which compounded the lack of sustained elimination [38, 39].

The low level of community participation and knowledge of schistosomiasis was quite unexpected, given the “good story Botswana wrote about herself” concerning the past control strategies. In addition, this finding was contrary to previous studies in other schistosomiasis-endemic regions. In the past for instance, Ndamba and others reported [40] that 80% of villagers in Zimbabwe were aware of schistosomiasis, whereas other studies in Egypt [41] and Brazil [42] revealed that people were fairly familiar with schistosomiasis. One explanation of this finding could be that integration of the health education program might have positively influenced the knowledge level of care providers, access to health care, and healthcare-seeking behavior of the community. Bizimana and colleagues [34] in their review found no positive effect of integration on the knowledge level of symptoms and modes of transmission at the community level. Another explanation for the low level of community engagement could be the partial centralization of disease control services. This process of partial decentralization made the program struggle to find adequate support from the districts.

While it is undeniable that SAC is the most at-risk group, epidemiological evidence has pointed out the need for the inclusion of other groups (pregnant women, pediatrics, and adult groups) [43, 44] in the control program. The 2015–2020 master plan lacked policy details on how the aforementioned groups will be included. It is also interesting to note the omission of pediatrics in the recent (2022) national MDA campaign. Other far-reaching consequences in the area of policy process included the lack of prioritization of other key interventions of health education and behavior change as greater emphasis was placed on treatment. Our results resonate with Chimbari and colleagues who advocated for country-level experience and nontreatment measures in Zimbabwe [45]. Ownership of the policies by the main stakeholders in the implementation process and valuing the local context is deficient. This is revealed by the poor community participation and low levels of knowledge of the disease posed by communities in Ngamiland [46].

Our analysis showed a comprehensive and practical involvement of policy-makers as actors. The policy actors were not balanced and deficient in representation. It is worth noting that there were other actors in the district working with communities on the site or with projects in the area. Some of these actors, such as the NGO community, VDC, VHC, and local authorities had networked with donors working at the international level. The NGO community also helps in advocacy and lobbying at the grassroots level. Although agencies like WHO have supported Botswana through technical resources and drugs, one of the major challenges in program implementation was the inadequate resources from the government. Key informants from the study complained about a one-man office and lack of domestic resources and too much reliance of the program on the TB program. In their policy analysis for newborn care, Ahamed and colleagues [47] alluded that the sharing of authority, resources, and decision-making, often leads to challenges of inadequate policy-making and poor governance for the program which in turn impairs the control of the disease. We note that poor governance impairs the health system and the ability of the decision-makers to develop and implement key policies for the control of schistosomiasis.

This study has some limitations**.** First, a small sample size and the online and telephone interviews may have deprived the researcher and the interviewees of an opportunity for further probing and questioning that normally occurs in a face-to-face interview. However, it is worth noting that, the sample was adequate since data saturation was reached when further interviews yielded no new ideas. Another limitation of this analysis was using the Ngamiland health policy documents and plans without the inclusion of other districts, limited comparison of policy issues like integration and decentralization of services. However, we could only access documentation from Ngamiland which had a historic track role in controlling the disease.

## Conclusions

In this study we observed that the health policies and strategies of Botswana including those of the Ngamiland district did not include preschool-aged children in the preventative therapy and strategies for treating foci areas such as the Ngamiland region. In addition, the control policy remains silent on the guidance of addressing dynamic environmental transmissions and snail control. Moreover, the NTD program suffered lack of autonomy and lack of resources because it relied on the TB program. Further, devolution of authority and resources from the TB program is required, and districts need to reorient the NTD guidelines according to the burden of schistosomiasis morbidity, local culture, and healthcare system context. There is a need to develop national guidelines on treating foci of intense transmission despite the good coverage of preventative therapy. This is important as the country revitalizes the program.

### Supplementary Information

**Additional file 1**. Appendix 1: Consolidated criteria for reporting qualitative research (COREQ).

## Data Availability

The data set used to support the findings of this study is available from the corresponding author upon request.

## Abbreviations

COVID-19: Corona Virus Disease of 2019
DHMT: District Health Management Team
HPT: Health policy triangle
MDA: Mass Drug Administration
M&E: Monitoring and evaluation
MOH: Ministry of Health
NTD: Neglected tropical disease
NGO: Non-Governmental Organization
PTF: Policy triangle framework
SAC: School-aged children
SSA: Sub-Saharan Africa
STH: Soil-transmitted helminths
UN: United Nations
VCMS: Vector Control Management System
VDC: Village Development Committees
VHC: Village Health Committee
WHA: World Health Assembly
WHO: World Health Organization
