# Supplementary material for: A mixed method analysis of the Botswana schistosomiasis control policy and plans using the policy triangle framework
Source: Glob Health Res Policy. 2023 Sep 6;8:39. doi: 10.1186/s41256-023-00321-2 (PMC10481564; doi:10.1186/s41256-023-00321-2)
Supplement: Supplementary file 1 — Additional file 1. Appendix 1: Consolidated criteria for reporting qualitative research (COREQ). [file 41256_2023_321_MOESM1_ESM.docx]

**Appendix 1:** Consolidated criteria for reporting qualitative research (COREQ)

| **No** | **Item** | **Guide questions/description** | **Responses** |
| --- | --- | --- | --- |
| **Domain 1: Research team and reflexivity** | | |  |
| Personal Characteristics | | |  |
| 1. | Interviewer/facilitator | Which author/s conducted the interview? | **GPK** conducted interviews |
| 2. | Credentials | What were the researcher's credentials? *E.g. PhD, MD* | PhD student |
| 3. | Occupation | What was their occupation at the time of the study? | Researcher |
| 4. | Gender | Was the researcher male or female? | Female |
| 5. | Experience and training | What experience or training did the researcher have? | Trained and had conducted qualitative interviews before |
| Relationship with participants | | |  |
| 6. | Relationship established | Was a relationship established prior to study commencement? | Yes, during introductions |
| 7. | Participant knowledge of the interviewer | What did the participants know about the researcher? e*.g. personal goals, reasons for doing the research* | Though reasons for doing the research |
| 8. | Interviewer characteristics | What characteristics were reported about the interviewer/facilitator? | *reasons and interests in the research topic* |
| **Domain 2: study design** | | |  |
| Participant selection | | |  |
| 9. | Sampling | How were participants selected? | purposive |
| 10. | Method of approach | How were participants approached? | face-to-face, zoom and telephone, |
| 11 | Sample size | How many participants were in the study? | Twelve |
| Setting | | |  |
| 12. | Setting of data collection | Where was the data collected? | Zoom calla |
| 13. | Description of sample | What are the important characteristics of the sample? | Described in table 2 |
| Data collection | | |  |
| 14. | Interview guide | Were questions, prompts, guides provided by the authors? | Yes |
| 15. | Audio/visual recording | Did the research use audio or visual recording to collect the data? | Audio recording used |
| 16. | Field notes | Were field notes made during and/or after the interview? | Yes |
| 17. | Duration | What was the duration of the interviews? | Described under the methods sections (average 45 minutes) |
| 18. | Data saturation | Was data saturation discussed? | Described in the method section; Francis and colleagues` approach of data saturation. |
| **Domain 3: analysis and findings** | | |  |
| Data analysis | | |  |
| 19. | Number of data coders | How many data coders coded the data? | GPK coded the transcriptions and worked with PN, and DELP to refine and name themes. |
| 20. | Description of the coding tree | Did authors provide a description of the coding tree? | Codes based on the HPTF |
| 21. | Derivation of themes | Were themes identified in advance or derived from the data? | Derived from the data |
| 22. | Participant checking | Did participants provide feedback on the findings? | Three participants validated the findings |
| Reporting | | |  |
| 23. | Quotations presented | Were participant quotations presented to illustrate the themes / findings? Was each quotation identified? | Yes, participants identified by number and designation |
| 24. | Data and findings consistent | Was there consistency between the data presented and the findings? | Yes |
| 25. | Clarity of major themes | Were major themes clearly presented in the findings? | Yes, and elaborated by sub-themes |
| 26. | Clarity of minor themes | Is there a description of diverse cases or discussion of minor themes? | Yes |
